# P55 - Air-pollution and respiratory symptoms in children

**DOI:** 10.1186/2045-7022-4-S1-P110

**Published:** 2014-02-28

**Authors:** Zorica Živković, Sofija Cerović, Jasmina Jocić-Stojanović, Vesna Ivančević, Ivana Filipović, Ksenija Jevtić, Ljubica Marić

**Affiliations:** 1 MC "Dr. Dragiša Mišović "Children's Hospital for Pulmonary Diseases and Tuberculosis, Belgrade, Serbia; 2 Health Center Budva, Budva, Serbia; 3 US Medical School, Belgrade, Serbia

## Introduction

Studies of school environment and related health diseases in pediatric population have been performed recently. The European Commission, through the Directorate General for Health and Consumer Affairs, funded the study on Health Effects of School Environment held in different European countries. Levels of air pollutants can be several folds higher exposures are prolonged. Since children spend a large part of the day in school environment, nationwide initiatives to evaluate such indoor air quality (IAQ) were developed.

## Material and methods

The study protocol includes: one standardized questionnaire on school characteristics and IAQ policy completed by teachers, two standardized questionnaire derived from the International Study of Asthma and Allergy in Childhood questionnaire o n characteristics of children one filled in by the pupils and the other by their parents, school environment assessments and no n invasive clinical tests.

## Results

Previous studies revealed that pupils exposed to an elevated level of indoor PM10 and CO2 showed higher prevalence of all respiratory disorders than those exposed to lower level, significantly so for dry cough and as regards CO2, also for rhinitis. The prevalence of dry cough significantly (p,0.001) decreased with decreasing mean indoor levels of PM10 and CO2.

